# Extracellular vesicles report on the MET status of their cells of origin regardless of the method used for their isolation

**DOI:** 10.1038/s41598-020-75817-9

**Published:** 2020-11-04

**Authors:** Zivile Useckaite, Anindya Mukhopadhya, Barry Moran, Lorraine O’Driscoll

**Affiliations:** 1 School of Pharmacy and Pharmaceutical Sciences and Trinity Biomedical Sciences Institute, Trinity College Dublin, Dublin 2, Ireland; 2 School of Biochemistry and Immunology, Trinity Biomedical Sciences Institute, Trinity College Dublin, Dublin 2, Ireland; 3 Trinity St. James’s Cancer Institute, Trinity College Dublin, Dublin 2, Ireland

**Keywords:** Cancer, Cell biology

## Abstract

MET pathway is an important actionable target across many solid tumour types and several MET inhibitors have been developed. Extracellular vesicles (EVs) are proposed to be mini-maps of their cells of origin. However, the potential of EVs to report on the MET status of their cells of origin is unknown. After applying three proposed methods of EV separation from medium conditioned by three cell lines of known MET status, this study used an extensive range of methodologies to fundamentally characterise the resulting particles (nanoparticle tracking analysis, TEM, flow cytometry, immunoblotting) and their MET status (RT-qPCR and ELISAs). The results indicated that ultracentrifugation on density-gradient (UC-DG) consistently produced the most reliable data with regards to purest EVs. EV cargo reflected MET mRNA, total MET and pMET status of their cells of origin. In conclusion, to simply determine if the general contents of conditioned medium reflect the MET status of the conditioning cells, choice of method for initial EV separation may not be crucial. However, to be confident of specifically studying EVs and thus EV-MET cargo, UC-DG followed by extensive EV characterisation is necessary.

## Introduction

Mesenchymal–epithelial transition factor (MET) is a receptor tyrosine kinase oncogenic driver in some non-small cell lung cancers (NSCLC)^1^, as well as in sub-groups of other cancer types^2–5^. Abnormal stimulation of multiple signalling transduction pathways downstream of MET promotes many of the hallmarks and challenges associated with cancer. For example, it is through proto-oncogene tyrosine-protein kinase SRC that MET acts as an epithelial-mesenchymal transition (EMT) promoter^6,7^. Aberrant activation of MET can occur through several mechanisms. These include *MET* amplification and higher gene copy numbers, as well as MET dysregulation through MET mutations. These which increase MET stability and accumulation on the cell surface and, thus, a targetable alteration. MET stimulation induces phosphorylation of specific tyrosine residues which, in turn, activate multiple downstream signalling pathways.

**Figure 1:**
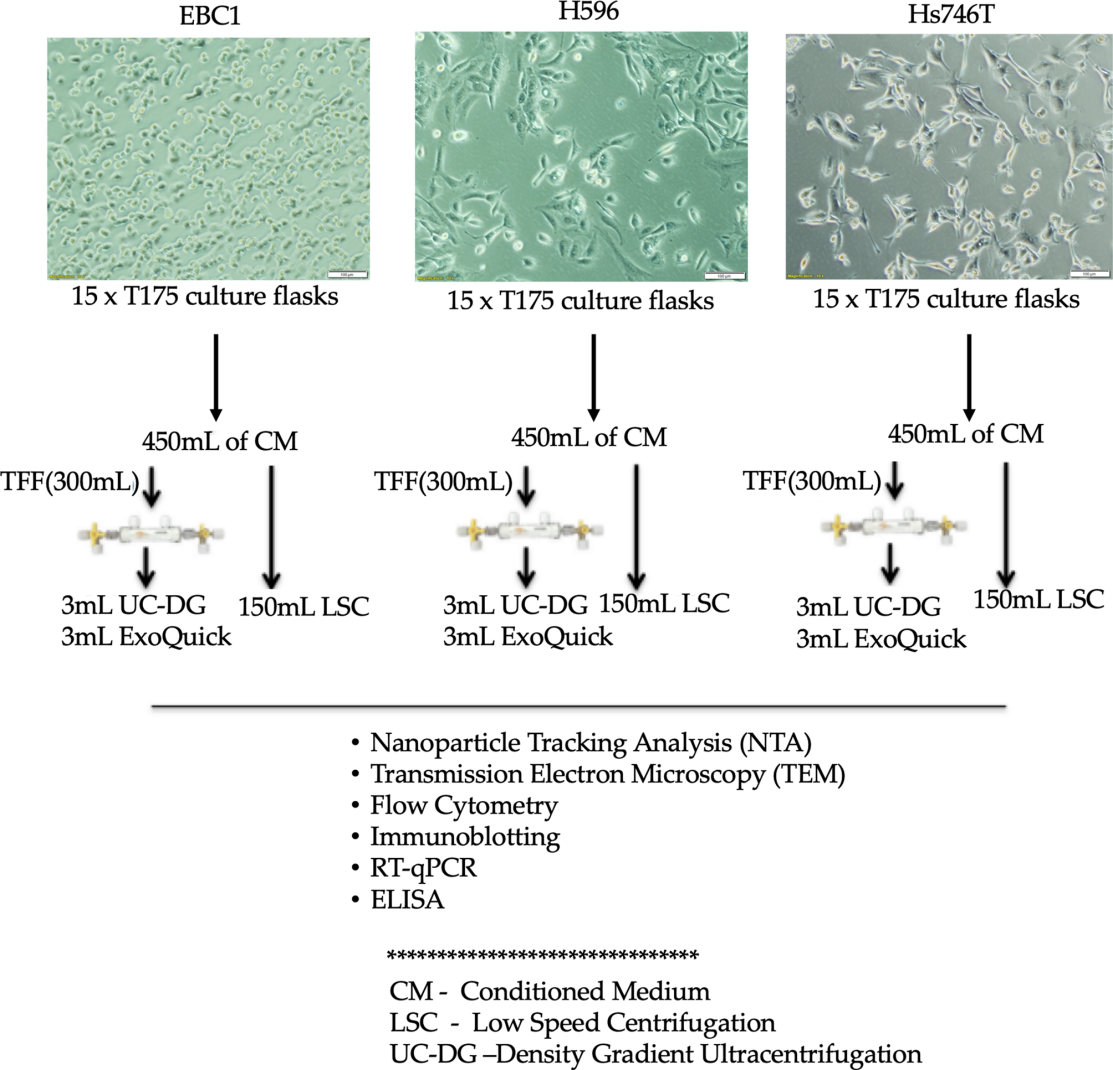
Experiment design. EBC1, NCI-H596 [H596], and Hs746T cells were seeded and allowed to attach overnight and then their appropriate medium was replaced with medium containing EV-depleted FCS. After 48 h, cell conditioned media (CM), was collected and cells were counted (to calculate the number of EVs released by a given number of cells, used for normalisation). CM from each cell line was pooled (i.e. 30 mL CM from fifteen T175 cm^2^ culture flasks of any given cell line, to produce each cell line’s pool of CM). Each 450 mL of CM was then divided into three equal parts and used for the three comparative methods of EV separation. The 150 mL of CM used for EV separation by ultracentrifugation on density gradient (UC-DG) and for ExoQuick was concentrated to approximately 3 mL each using tangential flow filter (TFF). The other 150 mL was used directly for low speed centrifugation (LSC). Following application of the 3 methodologies for EV separation, a range of characterisation was performed. These included nanoparticle tracking analysis (NTA) to determine EV concentration/yield, transmission electron microscopy (TEM) for EV size estimations and morphology; flow cytometry for EV surface marker characterisation; immunoblotting for lysed EV (exosomal and microvesicular) markers analyses^16^; RT-qPCR to detect MET; and ELISA to evaluate MET/pMET protein. This set-up was performed n = 3 independent times. EV-TRACK ID: EV200001.

The MET pathway is an important actionable target and so several MET inhibitors have been developed. These drugs can offer substantial benefit for treatment of several cancers where previously very few treatment options were available. However, only sub-groups of patients with these cancers can hope to benefit from MET inhibitors. Specifically, in up to 7% of NSCLC, *MET* amplification is an oncogenic driver^8,9^ and MET over-expression occurs in ~ 72% of NSCLC^10^. Companion diagnostics, particularly those that are minimally-invasive such as in the blood, are urgently needed for patient stratification and ease of selecting patients who have a chance of benefiting from MET inhibitors. Research, by our group and others, has strongly implicated extracellular vesicles (EVs) as minimally-invasive packages of biomarkers; if due consideration is given to appropriate pre-analytical sample preparation^11^. Many methods for EV separation from biological samples have been proposed. The most commonly used is ultracentrifugation, typically with density-gradients^12^. This method can result in quite pure EV preparations, but it is time-consuming, requires high-end equipment, and is not translatable to a routine clinical diagnostic setting. Some methods are simpler and less laborious, but purity of EVs is often compromised^13^. In the context of considering EVs as sources of biomarkers reporting on MET status of their cells of origin, here we evaluated a number of EV separation methods, that to the best of our knowledge have never been directly compared. We have then further investigated if EVs carry MET information representative of their cells of origin. Specifically, we have investigated the presence of the gene transcripts encoding for MET (MET mRNA), total MET protein, and activated/phosphorylated MET protein. The experimental design for this study is summarised in Fig. 1.

## Results

### UC-DG method of EV separation results in highest yields of EVs when compared to LSC or ExoQuick

**Figure 2:**
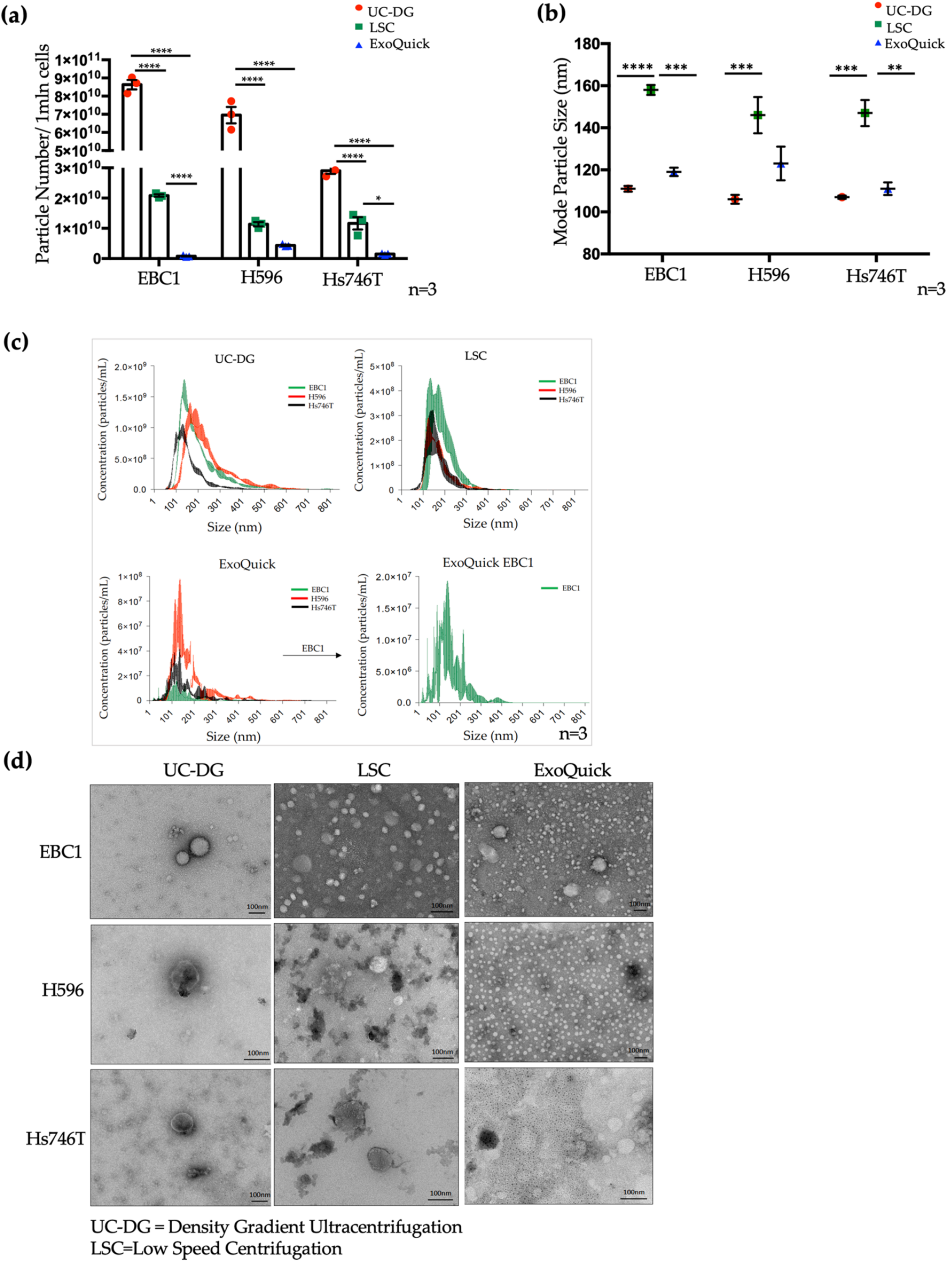
Quantification of EVs/particles by nanoparticle tracking analysis (NTA). (**a**) Quantification of EV particle number separated from the three different cell lines CM normalised to 1 × 10^6^ (denoted as 1 mln) of cells. Statistical analysis used ANOVA, where p < 0.05(*), p < 0.0001(****). Graphs represent data as mean ± S.E.M. of ≥ n = 3 repeat analysis on samples from n = 3 biological repeats. (**b**) NTA estimates mode particle size. Error bars denote mean ± S.E.M of ≥ n = 3 repeat analysis on samples from n = 3 biological repeats. (**c**) Raw NTA data showing particle range within each sample. Data for EBC1 could not be represented clearly when grouped with data from other cell lines’ EVs, due to not falling within the same range; therefore, presented separately. (**d**) Characterisation of EVs separated by UC-DG, ExoQuick and LSC using transmission electron microscopy (TEM). Scale bar = 100 nm.

For all cell lines’ conditioned media (CM) analysed, the highest particle counts (considered as particles/10^6^ cells) recorded by nanoparticle tracking analysis (NTA) was obtained by UC-DG method of EV separation; low speed centrifugation (LSC) produced the second highest quantities, followed by ExoQuick (Fig. 2a, Supplementary Table 1). EV samples were found to be heterogenous and particle populations differed (based on particle size distribution) depending on the isolation method (Fig. 2b,c). The most reproducible data (i.e. smallest errors bars) was obtained from the n = 3 independent repeat growths of cells and collection of EVs when UC-DG was used.

### UC methods produce cleanest EV isolates with the expected morphology of EVs

Representative images from TEM analysis (Fig. 2d, Supplementary Fig. S1) also showed that the size and heterogeneity of EVs/particles isolated depended on the separation technique employed. Specifically, TEM indicated that EV populations obtained by UC-DG had the least background debris and the majority of EVs were 40–140 nm in size. LSC produced EVs predominantly of ~ 40–150 nm, with some larger structures. ExoQuick isolates were very small (10–30 nm) particles. The EVs from UC-DG were round and appeared intact. This was not always so with the other methods.

### EV surface proteins are cell line-specific, rather than EV isolation method-specific

**Figure 3:**
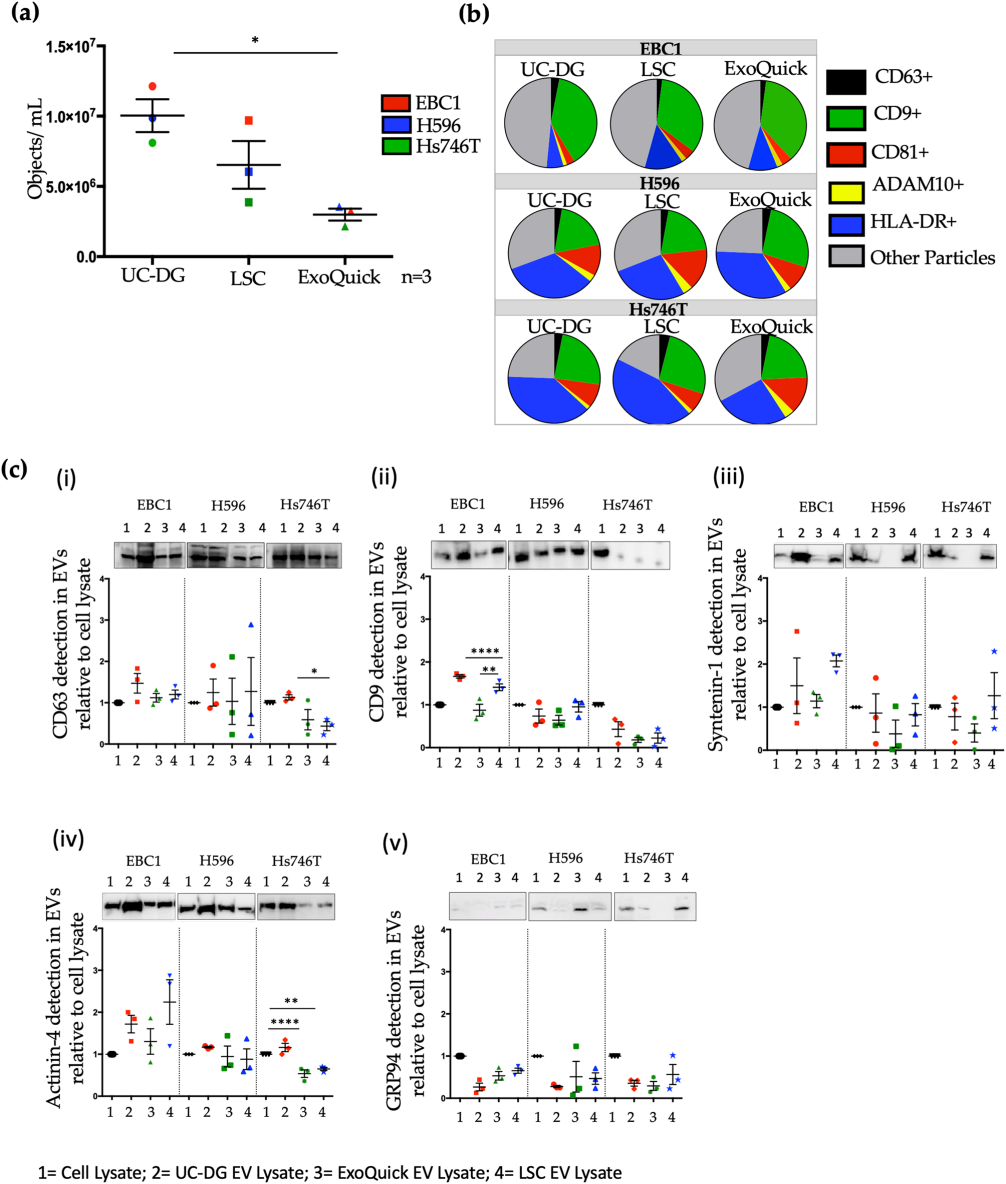
Analysis of single EVs by IFCM and analysis of some of total EV population by immunoblotting. (**a**) Quantification, by IFCM, of EVs per mL of sample isolated from EBC1, H596 and Hs746T following EV separation by UC-DG, LSC or ExoQuick. Data is represented as mean ± S.E.M. of ≥ n = 3 repeat analysis, on samples from n = 3 biological repeats. ANOVA was used for statistical analysis; p < 0.05(*). (**b**) Pie-charts illustrating heterogeneity of EV populations from CM-derived EV samples from EBC1, H596 and Hs746T cells, but separated with three different methods of EV isolation. (**c**) Immunoblot analysis of key proteins considered to be markers of EVs, according to MISEV2018 guidelines^16^. Along with other accepted EV markers, included here are tetraspanins CD63 and CD9 analysed used lysates of EV heterogenous pools, which were also evaluated as single intact EVs by IFCM.

Imaging flow cytometry (IFCM) was used to detect surface proteins on single EVs (objects). As with NTA results, by IFCM-across all cell lines-, total EV concentration was higher following EV separation by UC-DG, when compared to LSC and ExoQuick (Fig. 3a). Tetraspanins CD9, CD63, CD81, HLA-DR and ADAM10 were detected with all samples. A relatively high percentage of EBC1-derived EVs had surface CD9. Depending on EV isolation method applied, 33–38% EVs were CD9-positive (Fig. 3b, Supplementary Table 2) compared to 19–27% and 21–26% for H596 EVs and Hs746T EVs, respectively. Conversely, more H596-derived EVs and Hs746T-derived EVs displayed HLA-DR (27–34% and 26–44%, respectively) when compared to EBC1-derived EVs (6–14%) (Fig. 3b, Supplementary Table 2).

### Common EV markers detected in all EV/isolate lysates, with lowest levels following ExoQuick

Irrespective of EV separation method, common EV markers were detected by immunoblots in all EV lysates (Fig. 3c, Supplementary Figs. S2, S3), although differences in amounts were observed. For all, a representative immunoblot is presented, below which is a graphical representation of the densitometry performed on all 3 independent immunoblots. Of note, in the graphs, the densitometry values for the cell lysates was set at an arbitrary value of 1. Tetraspanins CD9 and CD63, as well as syntenin-1, are typically considered to be associated with exosomes. CD63 was detected with lysates from all cell lines and their EVs. The graphical representation of its densitometry analysis showed substantial variability between independent immunoblot for CD63. This variability was contributed to by the background “smeared” appearance that typically occurs with CD63 and which cannot be very accurately quantified by densitometry. In contrast, results for CD9 were very consistent between repeat experiments. EVs released by Hs746T cells were very low in CD9-positivity compared to EVs from EBC1 and H596 cells. Syntenin-1 was observed in isolates from all cell lines, but differences were found depending on the EV separation method; with ExoQuick isolates quite consistently showing low levels of this protein. Markers indicative of microvesicles (by actinin-4 detection) were found with all cell lines and some EV isolates from each cell line. However, substantial differences in actinin-4 positivity were observed, depending on the EV isolation method used. Traditionally, GRP94 was commonly used as a negative control for EVs, to confirm purity of sample. However, low levels of GRP94 were also detected, particularly following ExoQuick for H596 CM and LSC for Hs746T CM, suggesting non-EV contamination (see also Supplementary Figs. S2, S3).

### MET gene transcripts were detected in all cells and their released EVs

**Figure 4:**
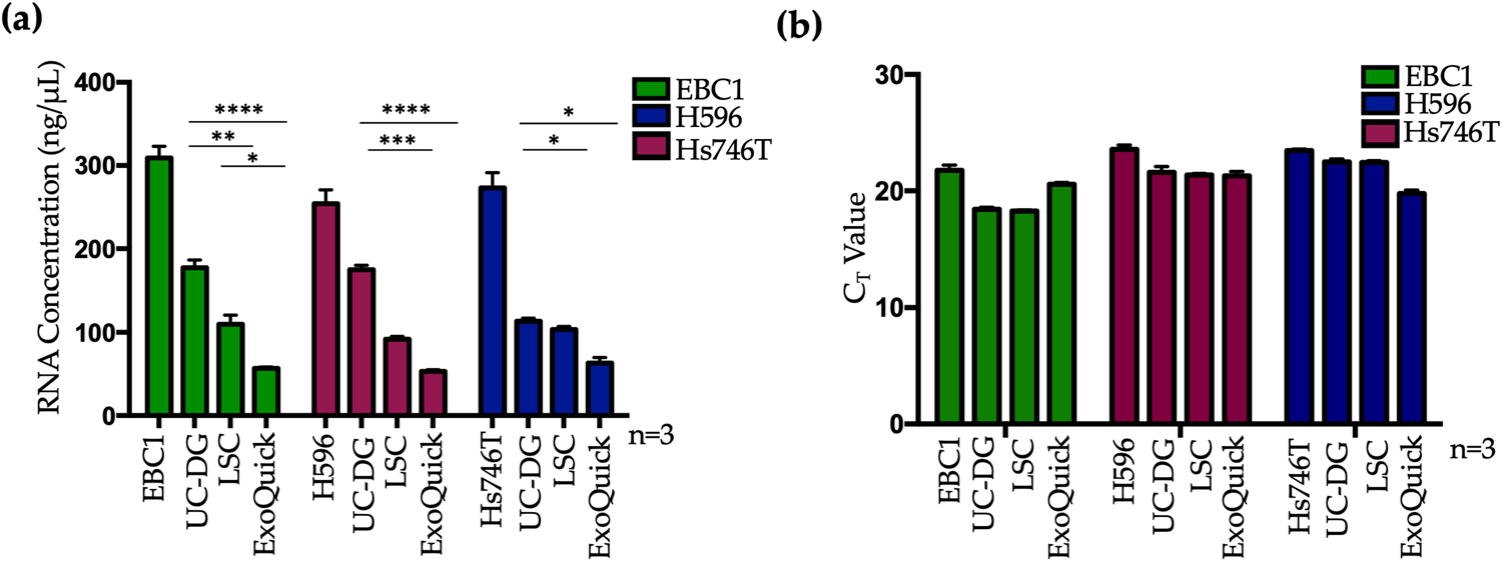
Detection of MET mRNA by RT-qPCR. (**a**) RNA concentration (ng/μL) in RNase-free water; (**b**) RT-qPCR measurement of MET mRNA in EBC1, H596 and Hs746T cells and their EV isolates. Graphs represent mean ± S.E.M. repeat analysis on samples from n = 3 biological repeats for MET gene transcripts. Statistical analysis was by ANOVA, where p < 0.05(*), p < 0.01(**), p < 0.001(***), p < 0.0001(****).

The highest concentration of RNA (ng/μL) was observed in EV samples from UC-DG, followed by LSC, then ExoQuick (Fig. 4a, Supplementary Table 3). As expected, higher RNA concentrations were obtained from cells compared to EVs. However, the starting material (i.e. cells vs EVs) cannot be considered as the same, so cell-derived RNA concentrations/yields are presented as a reference only to show the EV RNA data, which was of primary interest.

By RT-qPCR, MET was detected in EBC1, H596 and Hs746T cells and their released EVs following separation by UC-DG, ExoQuick, and LSC (Fig. 4b). Importantly, a minimum amount of RNA needed to be included for the RT and qPCR primers to work efficiently. Thus, 50 ng/µL of EV-RNA was used (i.e. 100 ng/reaction). In all cases where MET was detected in the cells, it was also detected in their corresponding EV isolates. However, the fact that UC-DG EVs carried more RNA than LSC EVs which, in turn, carried more RNA than ExoQuick isolates, means that the latter were “over-compensated” by the necessity to use a fixed starting amount of RNA.

### ExoQuick isolates carry higher quantities of MET and pMET, compared to those obtained with centrifugation methods

**Figure 5:**
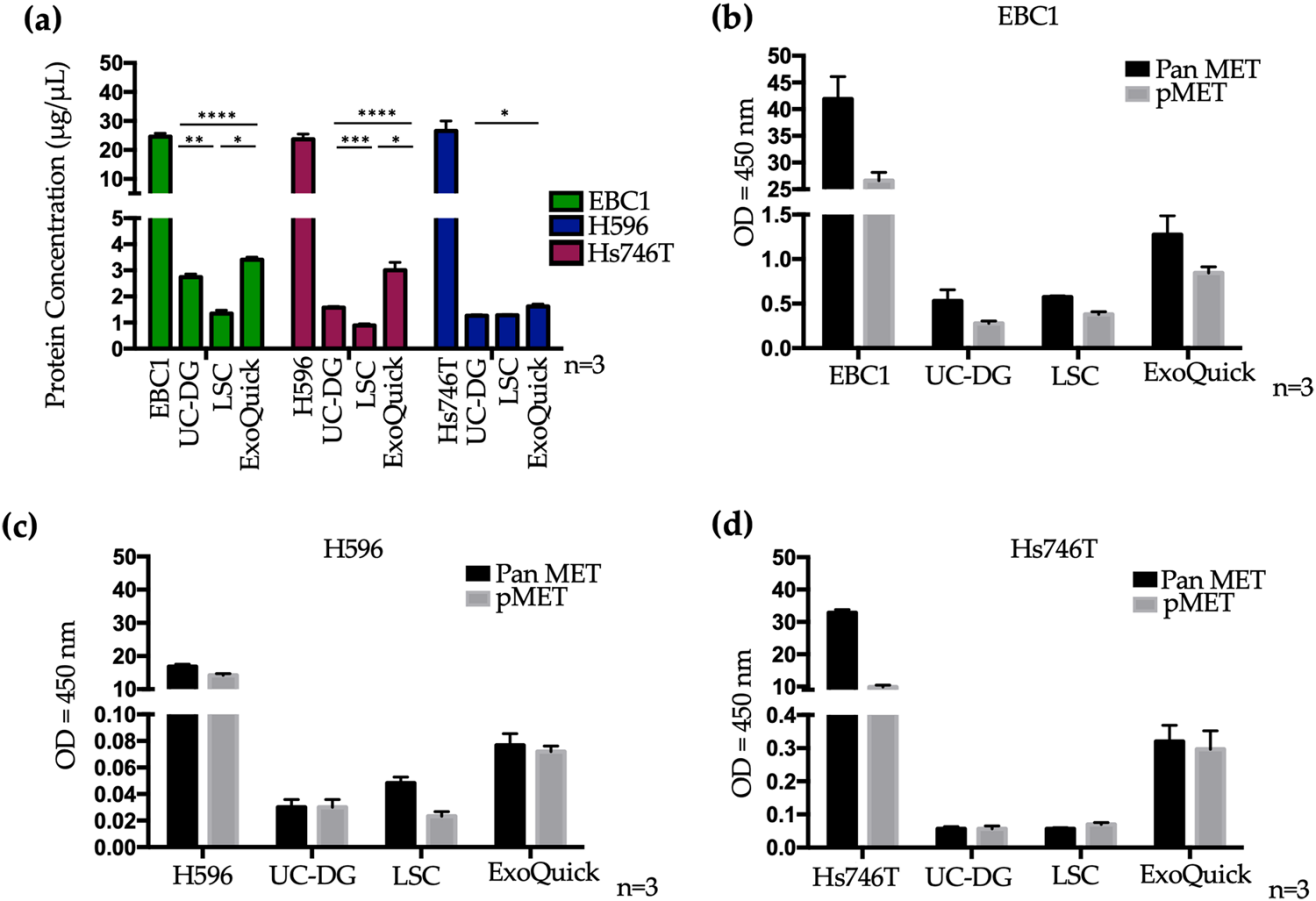
Total MET and phospho-MET detection by ELISA. (**a**) Protein concentration (μg/μL) and (**b–d**). Pan MET (total MET) and phosphorylated MET (pMET; Tyr 1234/1235) were detected for all cell lines and their EVs. All graphs represent mean ± S.E.M. of ≥ n = 3 repeat analysis on samples from n = 3 biological repeats. Statistical analysis was by ANOVA, where p < 0.05(*), p < 0.01(**), p < 0.001(***), p < 0.0001(****).

Total protein obtained from each cell line and each EV isolation method are shown in Fig. 5a (Supplementary Table 4). The highest yield of protein was measured following ExoQuick, followed by UC-DG, and then LSC. Higher yields of protein were, of course, obtained from cells than from EVs. Thus, the cellular starting material was not directly comparable to EVs, but used as reference only.

Using fixed quantities of cellular or EV total protein for all samples (as required for the ELISAs), measurement of total MET (Pan MET) and phospho-MET (pMET; Tyr 1234/1235) for cell and EV lysates (following the three different methods of EV separation) were performed. These showed that although cell lysates had higher amounts of total MET and pMET (Tyr 1234/1235) compared to EVs, all EV isolates carried some MET and pMET (Fig. 5b–d). Interestingly, ExoQuick isolates appeared to contain the highest quantities of both total MET and pMET, when compared to purer EV preparations achieved by UC-DG.

## Discussion

MET inhibitors show great promise for cancer treatment where there is MET amplification/over-expression and activation. This is particularly important as MET amplification/over-expression and activation, when present in cancer patients’ tumour tissue, have been associated with poor overall survival. Minimally-invasive companion diagnostics are urgently needed for patient stratification and ease of selecting patients who have a chance of benefiting from such drugs. As explained previously, MET has been well recognised as an actionable target in cancer. Indeed, many drugs have been developed for this purpose. However, not all tumours contain these actionable targets. For example, it has been reported that MET over-expression occurs in ~ 72% of NSCLC (but not in the other 28% of NSCLC). Thus, a precision medicine approach is necessary so as to not waste precious time of a patient’s life by administering an anti-cancer treatment from which that individual could not gain benefit; but would still endure its side-effects. Thus, the typical way of identifying those patients who would be candidates for such targeted anti-cancer treatment is to evaluate MET expression/over-expression and activation in tumour tissue samples. However, many tumours, e.g. those in the lungs, are not easily accessible in order to evaluate the solid tissue (e.g. by immunohistochemistry) for this purpose. Thus, if an entity released by the tumour cells into blood reflects the MET status of the tumour, this would have potential as a minimally-invasive means of predicting those individuals whose tumours are likely versus unlikely to respond to MET-targeted treatment.

Indeed, EVs in blood are showing promise as carriers of minimally-invasive biomarkers in cancer. However, for each given clinical indication and drug/drug family the relevance of EVs as biomarkers must be established. Thus, here we must establishing if EVs could inform on the MET/pMET status of their cancer cells of origin; which method(s) would be most suitable for isolating EVs for this purpose; and if EV-MET/EV-pMET could be reliably, robustly and reproducibly evaluated in a way that is translatable to the clinical [such as enzyme-linked immunosorbent assay (ELISA)], rather than only via fundamental laboratory procedures (such as immunoblotting).

Very few studies have reported on either MET or pMET in relation to EVs. In one study of melanoma, EVs were reported to increase the metastatic behaviour of primary tumours in pre-clinical models, by “educating” bone marrow progenitor cells^14^. Subsequently, however, the Reproducibility Project-intending to replicate this work-reported that they were unable to reliably detect pMET in exosomes^15^. Thus, no conclusive assumptions can be made as to whether or not MET and pMET are carried by EVs, from such opposing reports (which incidentally used immunoblots, not clinically-relevant ELISAs).

We believe this to be the first study extensively evaluating and comparing methodologies for EV separation and characterising from cells (including both lung cancer cells and gastric cancer cells) of known MET status, to establish the optimal method for EV separation and also to determine how the resulting EVs reflect the MET and pMET status of their cells of origin.

MISEV2018 guidelines highlight the importance of standardisation of sample collection, EV separation, and EV characterisation methods, to obtain meaningful data^16^. Of course, there are pros and cons for every method of EV separation^17,18^. In efforts to remove contaminating non-vesicular particles, ultracentrifugation on density-gradient has been shown to increase stringency, compared to other methods, by separating particles of different densities^19^. More recent published studies have also demonstrated that reasonably pure EV preparations, from plasma, can be separated by ultracentrifugation on iodixanol (OptiPrep) density-gradient (UC-DG). This method is proposed to produce higher yields of EVs than previously described separation techniques, without lipoprotein and albumin contamination^20^. Increasing numbers of commercially-available EV separation kits claim a quick and precise EV separation method, that could be used in the clinical setting. However, published studies suggest that commercial kits such as ExoQuick and total exosome separation, provide a higher extraction efficiency of general isolates than traditional UC, but that EV samples separated by UC are purer^21^.

In this study, we have shown by NTA and IFCM that the highest yield of EVs were separated from CM using UC-DG, indicating that, of the three methods compared, UC-DG is superior for most comprehensive recovery of EV. NTA also analysis suggested that particles separated by ExoQuick are the smallest, with more heterogenous EV populations and particle sizes measured in EV samples separated by UC-DG. As expected, particles isolated by LSC were the largest (based on mode particle size). It has been suggested that ExoQuick may be most suitable where the population consists of mainly small EVs. However, it must also be considered that the yield of EVs was so much lower with ExoQuick than with the other methods evaluated**.** This indicates that a large percentage of the heterogeneous EV population was lost. This may bias the outcome of a study i.e. it may give us the impression that the population consists of mainly small vesicles, but that may be because the rest of the vesicles have been lost by this process. Of course, we feel it is important to reiterate that no method for EV is ever likely to successfully collect all EVs.

EV characterisation by transmission electron microscopy (TEM) also showed that the apparent size and heterogeneity is dependent on the EV separation technique applied to each cell line’s CM. TEM images also suggest that the UC-DG method produces the purest EVs with the lowest levels of background material^20^. TEM indicated that the majority of EVs separated by UC-DG were 40–140 nm in size. As predicted, and in line with NTA findings, some of the largest EVs, up to 200 nm, were separated by LSC. ExoQuick isolated were typically very small (~ 10–30 nm), with extensive background material. Previously studies have reported that ExoQuick isolates particles may be linked to the aggregation and precipitation of other elements in any given sample, but that are not EVs per se. This claim was supported by data showing that the amount of residual proteins was significantly higher following ExoQuick compared to UC^22^. In keeping with this, in our study, ExoQuick isolates had the lowest EV/particle/object numbers per million donors cells (as shown by both nanoparticle tracking analysis (NTA) and by imaging flow cytometry **(**IFCM)); had the lowest RNA yields; and yet contained the highest protein levels, suggesting the presence of non-EV material.

IFCM was used to analyse surface EV protein markers on intact, individual EVs. By IFCM, consistent with the NTA data, total EV yield was found to be higher with UC-DG compared to LSC, and significantly higher when compared to ExoQuick. This was the situation with all three cell lines. All EV surface markers studied (CD63, CD9, CD81, ADAM10 and HLA-DR; selected from MISEV2018 guidelines) were detected in samples to varying concentrations. Overall, the IFCM data confirms the NTA data that EV populations separated from CM from any given cell line -but using different methods of EV separation-differ in yield, with UC-DG separating a significantly higher amount of EVs.

Immunoblotting was used to analyse EV protein markers on the lysed heterogenous pool of EVs (and cells). Immunoblotting confirmed the presence of EV markers (CD9-, CD63- and syntenin-1, and actinin-4) but, again, with differences depending on the cell line and the EV separation methods; in concert with studies on other cell lines/EVs^21^. As MISEV2018 guidelines indicate, we must continue to analyse a number of EV markers to establish if we have isolated EVs, as no one marker is likely to be common to all cells types heterogeneous EV populations, isolated using different methods. The detection of GRP94, *albeit* typically at relatively low levels, supports the finding by others that GRP94 is not necessarily a negative marker for all EVs. Its detection here with some cell lines’ isolates, particularly with ExoQuick isolates and LSC isolates—and so a lesser extent, UC-DG isolates—also indicates (that which we already suspected) that some of our EV preparation are purer than others.

It is noteworthy that different EV characterisation methodologies may be considered to give inconsistent data. However, because different types of analyses evaluate different aspects of EVs, it is typical that they do not produce exactly the same results. If that was so, some methodologies would be redundant. Thus, the intention of using a broad range of techniques for EV analysis is for added value and to get as comprehensive understanding of the EV population(s) in hand, as possible. This is a relatively new but expanding field, with no single type of analysis accepted for characterising of all aspects of EVs. There may never be. So, for example, considering CD63 and CD9 analysis by flow cytometry vs immunoblotting: for immunoblotting, lysed pools of EVs are studied; for IFCM, intact single EVs are studied. So, one method is evaluating single intact EVs, the other investigates the heterogeneous pool of EVs in a lysed state. Thus, the reason for using both techniques at this stage in EV research is to generate as complete an understanding as possible about the EVs. Similarly, for TEM vs NTA, these two complementary methodologies will not generate exactly the same results, as they are investigating EVs in different ways. NTA was used with the aim of estimating quantities and sizes all particles in the samples, whereas TEM was used to visually evaluate EV structure, size and sample background. So, although there is not full accordance, we believe that in a study that compares methodologies it is important to be as comprehensive with our characterisation as possible. It highlights the limitations of individual techniques and the need to continue using a range of techniques for fundamental characterisation of EVs.

With the objective of assessing how well EVs from each separation method represent the MET transcript status of their cells of origin, RT-qPCR and ELISAs were performed. Different yields of RNA were separated from EV samples obtained via different methods of EV isolation. However, for optimal use of RT and qPCR primers, a constant amount of RNA had to be used. This caused a bias towards the ExoQuick method of EV isolation. Specifically, there was less RNA available from ExoQuick isolates, but the same amount of RNA had to be used for RT-qPCR as for UC-DG and LSC. Due to the lack of reliable control RNA for EVs, quantification could not be performed. However, we can conclude that C_T_ values were compared, and all data was highly reproducible between repeats (technical and biological). Furthermore, MET mRNA was detected with all EV samples analysed, as well as in their cells of origin.

For the most part, EVs appear to reflect MET status of their cells of origin. As mentioned above, RT-qPCR showed the expression of MET in all cell types and their EVs, following EV separation using each of the three methods. The Hs746T results were as predicted, whereas the H596 results were unexpected, as these cells have been suggested not to harbour MET^23^. However, our stock of these cells was purchased directly from ATCC for this study, so we are confident of their pedigree. Crucially, however, what was observed in the cells was also observed in their corresponding EVs.

Total MET and phosphorylated MET proteins were also detected in all cell lines and all EVs, regardless of the EV separation method. However, the amounts detected in EVs were substantially less than in the cells. Overall, the highest levels of total MET and pMET were apparent in ExoQuick isolates, although these isolates were not rich in EVs (i.e. they had relatively low quantities of EV as evaluated by NTA and IFCM, and relatively low amounts of total RNA). It can be considered that, as TEM results indicate the ExoQuick preparations carry most impurities, it is possible that this larger amount of total protein and, more specifically, total MET and pMET is due to the presence membrane fragments; rather than only intact EVs.

Altogether, our data suggests that UC-DG, compared to ExoQuick and LSC, is the most efficient method to recover the highest yield of EVs from CM to produce the purest EV samples. Due to lengthy, labour-intensive protocols, UC-based methods are arguably not most suitable for diagnostic use in a clinical setting. However, they provide a valuable platform for quality EV separation in research. Our data suggests a potential use of commercially-available kits in the clinical setting, if pure EVs are not required. Overall, our studies showed that although, of course there was less MET mRNA, MET and p-MET proteins carried in EVs than in their cells of origin, the EV cargo reflected that observed for their cells of origin.

## Materials and methods

### Cell culture

Cell lines of differing MET status were included. EBC1 (Health Science Research Resources Bank) have MET amplification; NCI-H596 [H596] (ATCC) have mutated MET (i.e. an exon 14 skipping mutation); and Hs746T (ATCC), portrays both MET and mutated MET amplification (exon 14 skipping mutation). As recommended by the Suppliers of these cells, EBC1 were cultured in RPMI-1640 medium; H596 were cultured in RPMI-1640 medium; and Hs746T were cultured in DMEM). 10% (v/v) EV-depleted FBS was required for all lines. All experiments were done a minimum of 3 times, starting with a new vial of cells each time.

### Conditioned medium (CM) preparation

H596 and Hs746T cells were seeded at the density of 5 × 10^6^ cells/T175 cm^2^ culture flask in 30 mL of medium. EBC1 cells were seeded at 3.5 × 10^6^ cells/T175 cm^2^ culture flask in 30 mL medium. Twenty hours later, medium was replaced and conditioned for 48 h. CM (total = 450 mL) was centrifuged at 750*g* for 10 min (twice) and 2800*g* for 10 min (twice) to remove cell debris. Cells were counted to calculate EV numbers/releasing cells.

### Tangential flow filtration (TFF)

Tangential flow filter (TFF, HansaBioMed, Cat. #: HBM-TFF/1) was used to concentrate CM, exactly as per manufacturer’s recommendation. Each 150 mL of conditioned media (CM) was concentrated to 3 mL (i.e. concentrated by a factor of 50) for each cell line, prior to isolation using UC-DG and ExoQuick, respectively. Fresh TFF columns were used for each cell line to avoid cross-contamination. Each TFF column was re-useable up to 20 times, as per manufacturer’s recommendation.

### Ultracentrifugation on density gradient (UC-DG)

EVs, including small EVs, are expected to be isolated by UC-DG. OptiPrep was diluted with PBS to create density-gradient fractions of 5–40%. Density-gradient fractions (bottom-up) were layered over the sample and centrifuged at 120,000*g* for 18 h at 4 °C (SW 32.1Ti swing-bucket rotor, Beckman Coulter). Seventeen fractions (1 mL each) were then collected (top to bottom). To calculate each of the 17 fraction’s density, the refractive index (RI) of each was measured using a Pal-Ri refractometer (Atago). The refractometer was zeroed/blanked using 300 μL of distilled water. Then a 300 μL aliquot of each UC-DG fraction was placed on the refractometer and read. Based on the measured RI, the following equation was used to calculate the density, as density = 3.3411 × RI–3.4584. EV/particle concentration in each fraction were also measured by nanoparticle tracking analysis (NTA; NanoSight NS300 system, Malvern Technologies, UK). Samples within density fractions of 1.12–1.2 g/mL (which were, as expected, also found to contain the highest concentration of EVs were pooled (Fig. S4), were pooled, washed with sterile particle-free PBS and centrifuged at 120,000*g* at 4 °C (SW 32.1Ti swing-bucket rotor, Beckman Coulter) in order to pellet EVs and to remove OptiPrep. EVs were re-suspended in PBS or cell lysis buffer (50% in each).

### ExoQuick

ExoQuick-TC kit (ExoQuick-TC Ultra for Tissue Culture Media, System Biosciences, Cat. #: EQULTEA-20TC-1) was used as per manufacturer’s recommendation. Specifically, 5 mL of CM must be used for EV isolation using the kit. Therefore, 2 mL of basic culture medium (RPMI-1640 for CM from EBC1 and H596 cells; DMEM for CM from Hs746T cells) was added to CM concentrates that were obtained following TFF (as described above) in order to bring the total volume of CM to 5 mL. No other changes were made to the process recommended by the manufacturer of ExoQuick-TC.

### Low speed centrifugation (LSC)

Large EVs are expected to be isolated by low speed centrifugation (LSC). Here, these EVs were isolated from CM by adapting a previously published protocol^24^. Following the centrifugation at 750*g* for 10 min (twice) and 2800*g* for 10 min (twice) to remove cell debris, LSC of CM was performed using a 70Ti fixed-angle rotor at 10,000*g* for 1.5 h at 4 °C. Supernatant was discarded and the EV pellet was re-suspended in PBS or cell lysis buffer (50% in each).

### Nanoparticle tracking analysis (NTA)

Particle size distribution was determined by NTA configured with a 488 nm laser and a high sensitivity scientific CMOS camera. Samples were diluted (1:20–1:100) in PBS (Sigma, Cat.#:D8537-6X500ML) and analysed under constant flow conditions (flow rate = 50). Data was analysed using NTA 3.1.54 software.

### Transmission electron microscopy (TEM)

Samples were prepared by adapting a previously published protocol^25^. Briefly, 4 μL of sample in filtered PBS were fixed (10 min, RT) with 4% glutaraldehyde on carbon-coated grids (Ted-Pella B 300 M, Mason Technology, Cat.#:01813-F). Grids were washed 3 times (5 min) at room temperature (RT) with purified water. These were then contrasted with 2% phosphotungstic acid (10 min, RT), washed 3 times, and examined by JEOL 1011 TEM (JEOL LTD, Japan).

### Protein isolation from cells

Cell pellets were lysed in ice-cold lysis buffer (ThermoFisher; Cat. #: FNN0011) with added protease inhibitor cocktail (Complete protease inhibitor tablets, Roche) and phosphatase inhibitor cocktail (Halt, Thermo Scientific; Cat. #: 78420) on ice for 25 min. These were then centrifuged at 10,000*g* for 10 min. at 4 °C. Soluble protein was measured by BCA assay (Thermo Scientific, IL, USA). Representative details of protein quantification in Supplementary Fig. S6.

### Protein isolation from EVs

EVs obtained by UC and LSC were lysed as for cells. ExoQuick isolates were lysed by mixing equal volumes of ExoQuick elute and lysis buffer. All samples were incubated on ice for 25 min, centrifuged at 10,000*g* for 10 min. at 4 °C. Soluble protein was measured by BCA assay (Thermo Scientific, IL, USA). Representative details of protein quantification in Supplementary Fig. S6.

### Immunoblotting

Cell and EV protein (30 μg)—isolated as described above—was used for immunoblotting as we previously described^26^, except that 5% BSA/PBS containing 0.1% Tween 20 (PBS-T) was used. Primary antibodies from Abcam were anti-CD63 (Cat.#:ab68418; 1/1000 in PBS-T); anti-CD9 (Cat.#:ab92959; 1/1000 in PBS-T); anti-syntenin-1 (Cat.#: ab83690; 1/1000 in 5%BSA/PBS-T); anti-actinin-4 (Cat.#:ab41525; 1/1000 in 5% PBS-T); and anti-GRP94 (Cat.#:ab2791; 1/500 in 5%BSA/PBS-T). Secondaries from Cell Signalling were anti-mouse (Cat.#:7076; 1/1000 in 5%BSA/PBS-T) or anti-rabbit (Cat.#:7-74; 1/1000 in 5%BSA/PBS-T).

### Imaging flow cytometry (IFCM)

Imaging flow cytometry (IFCM) characterisation of EVs was adapted from a previously published protocol^27^. EVs were analysed using an Amnis ImageStream^X^ Mark II Flow Cytometer with the following antibodies, all from Biolegend: anti-CD63-FITC (CD63-FITC; 1:150, Cat.#:353006); CD9-PE (1:1500, Cat.#:312106); CD81-PE-Cy7 (1:150, Cat.#:349512); HLA-DR-BV421 (1:600, Cat.#:307636) and ADAM10-APC (1:150, Cat.#:352706)^17,26,27^. EV-free IFCM buffer, unstained EVs, single-stained controls and FMO (fluorescence-minus-one) controls were run in parallel. Data analysis used IDEAS v6.2 (Amnis/Luminex, Seattle). EVs were gated as SSC-low vs fluorescence, then as non-detectable brightfield (fluorescence vs Raw Max Pixel Brightfield channel) (Supplementary Fig. S5). Gated EVs were confirmed in the IDEAS Image Gallery.

### Quantitative reverse transcription PCR (RT-qPCR)

RNA from cells and EVs was isolated using PureLink RNA mini kit (BioSciensces, Cat.#:12183018A) following the manufacturer’s recommendation. Concentrations of cellular and EV RNA was measured using NanoDrop spectrophotometer and RNA purity was estimated using 260/280 ratio. The cellular and EV RNA yields are shown in Fig. 4. For the RT and qPCR steps, TaqMan MET assay and TaqMan reagents were used throughout following manufacturer’s instructions. In brief, cDNA was prepared using 16 µL RNA (50 ng/µL) and 2 µL (100 ng) were used per reaction. cDNA synthesis was performed using a DNAEngine Peltier Thermal Cycler (Bio-Rad Laboratories) to incubate samples for 10 min at 25 °C, 10 min at 50 °C followed by 5 min at 85 °C. qPCR was run in a ViiA7 Real-Time PCR System (Applied Biosystems).

### Enzyme-linked immunosorbent assay (ELISA)

Cell and EV protein samples (30 μg)—isolated as described above—were analysed using ELISA (Abcam, Cat.#:ab126451) for total MET and pMET (pY1234/1235). ELISAs were performed according to their manufacturer’s instructions.

### Statistical analysis

Unpaired two-tailed Student’s t-test was performed for comparisons between two groups. One-way ANOVA was used to compare more than two groups. Statistical analyses were performed using Graph Pad Prism 6 software. p < 0.05 was considered statistically significant*.*

## Supplementary information

Supplementary Information.
