# Supplementary material for: Extracellular vesicles report on the MET status of their cells of origin regardless of the method used for their isolation
Source: Sci Rep. 2020 Nov 4;10:19020. doi: 10.1038/s41598-020-75817-9 (PMC7642384; doi:10.1038/s41598-020-75817-9)
Supplement: Supplementary file 1 — Supplementary Information. [file 41598_2020_75817_MOESM1_ESM.pdf]

Supplementary Information

Title: Extracellular vesicles report on the MET status of their cells of origin regardless of the method used for their isolation

Authors: Zivile Useckaite[1], Anindya Mukhopadhya[1], Barry Moran[2] and Lorraine O'Driscoll[1,3]*

Affiliation: [1]School of Pharmacy and Pharmaceutical Sciences & Trinity Biomedical Sciences Institute, Trinity College Dublin, Dublin 2, Ireland; [2]School of Biochemistry & Immunology, Trinity Biomedical Sciences Institute, Trinity College Dublin, Dublin 2, Ireland; [3]Trinity St. James's Cancer Institute, Trinity College Dublin, Dublin 2, Ireland.

(a)

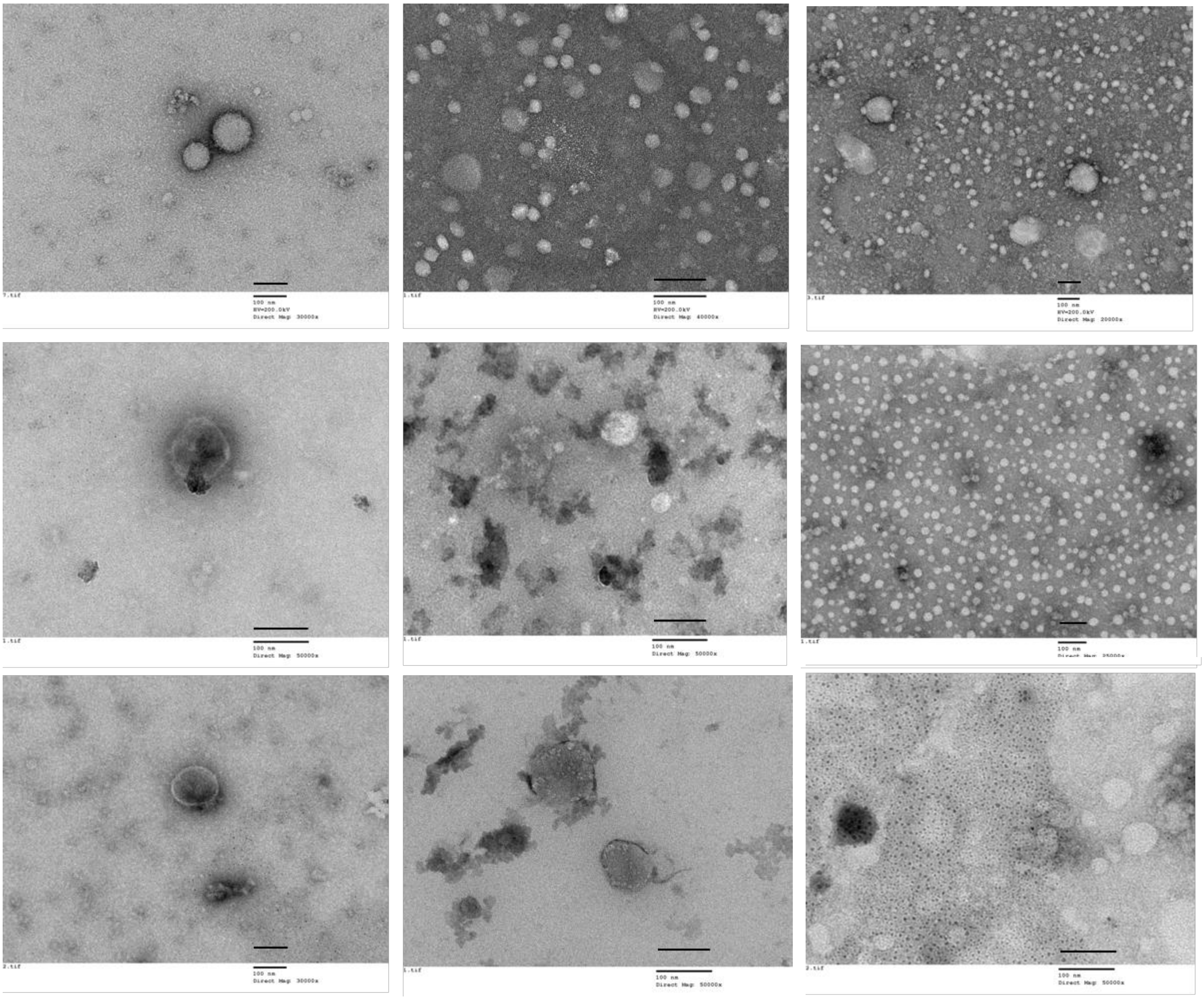

(b)

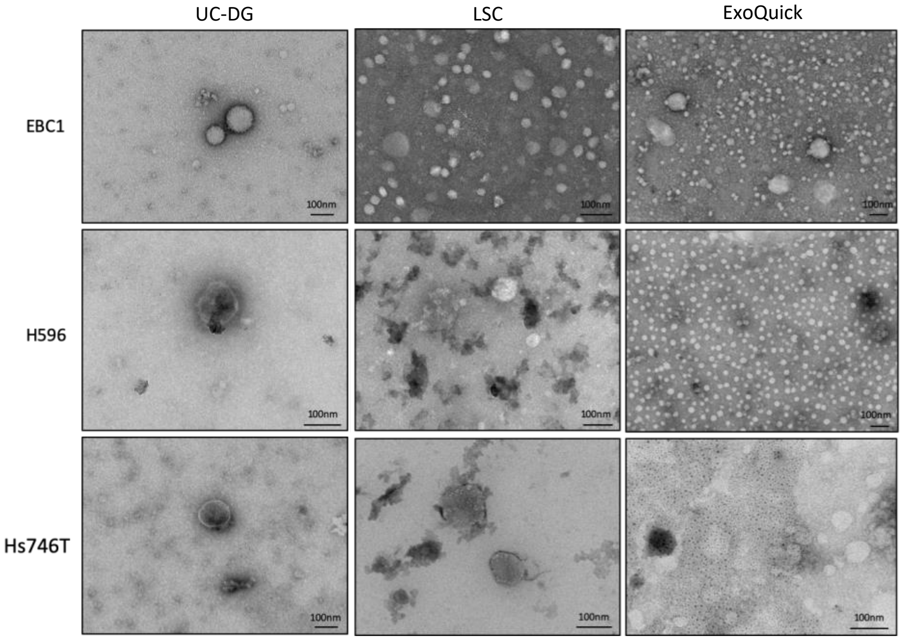

S1. Unedited TEM images in (a) and adapted for the paper in (b)

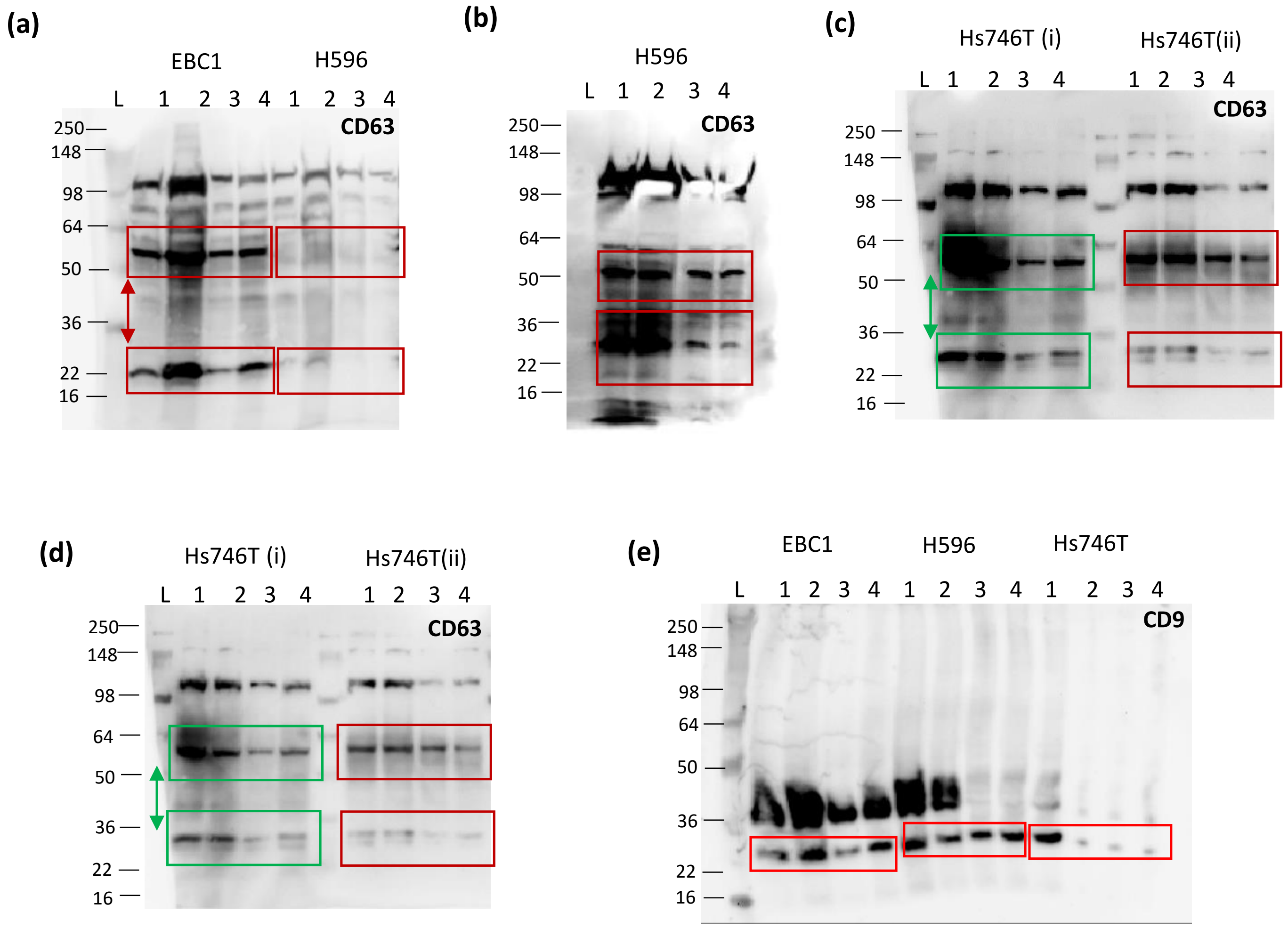

*Abcam reference. CD63 accepted band size between 30-60kDa.

*Abcam reference. CD9 band of approximately 22 kDa (predicted molecular weight: 25 kDa).

Additional note. At previous immunoblotting experiment (CD63 in Hs746T cell and EV lysates) no signal was detected. Experiment was repeated by loading 40μL of protein for reference/investigation, as seen in c, d as Hs746T(i) and the standard 30μL in Hs746T(ii). Bands from the additional run with 40μL of protein are boxed in green and are not used for the main text.

No signal was detected for CD63 in H596 cells. Based on the presence of Actinin-4 but the absence of CD63, H596 samples were run separately. Signal was detected after 1min exposure (in comparison to 15s for EBC1 and Hs746T).

S2. Images of non-cropped immunoblots from Figure 3c. Lane 1=Cell lysate, 2=UC-DG EV lysate, 3=ExoQuick EV lysate, 4= LSC EV lysate.

(a)

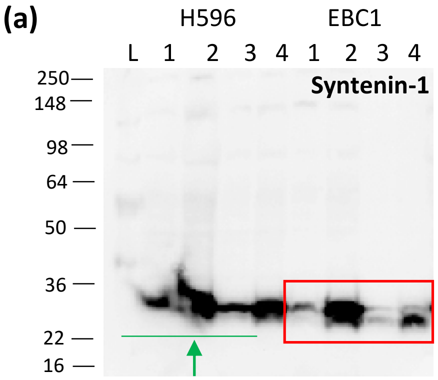

The gel did not run well on one side.
Experiment was repeated

(b)

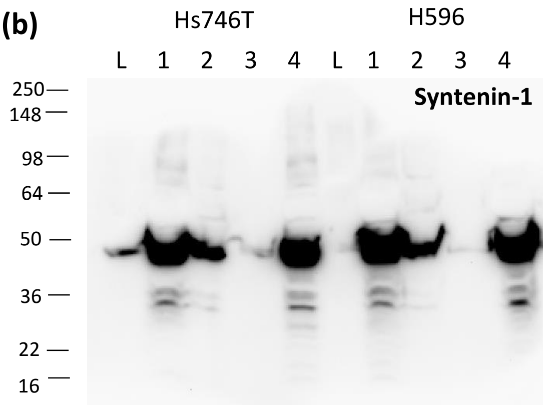

*Abcam reference. Predicted Syntenin-1 band size: 32 kDa. Additional bands are possible at 50kDa.

(c)

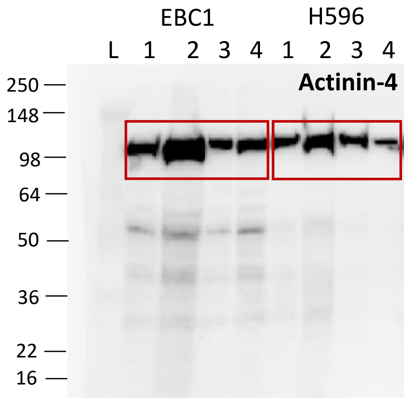

(d)

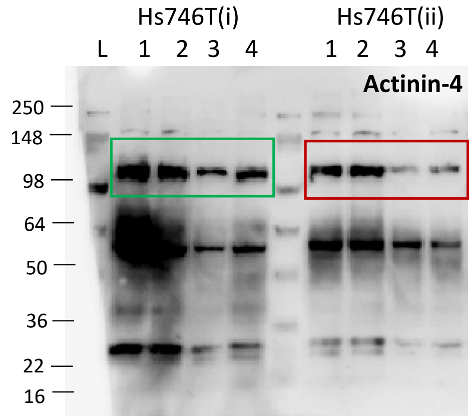

*Abcam reference. Predicted Actinin-4 band size: 105 kDa.

(e)

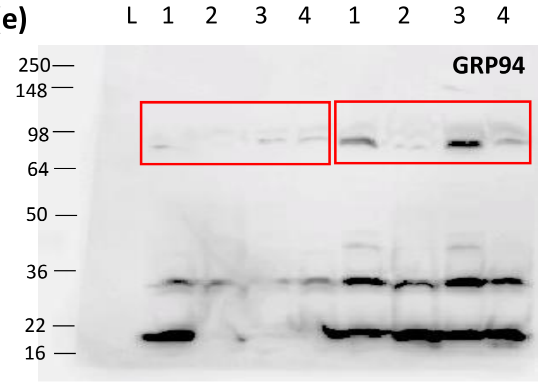

(f)

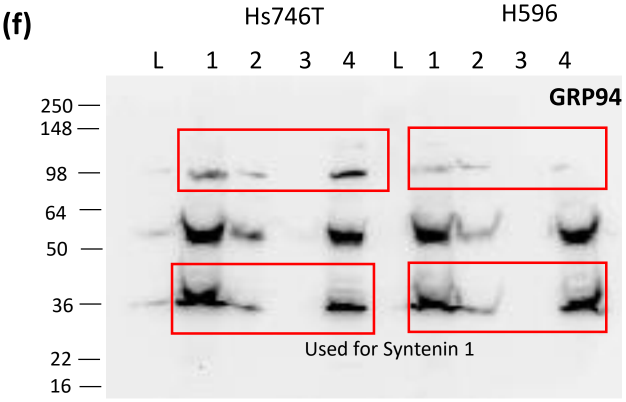

*Abcam reference. GRP94 two bands possible. The one to be used is at approx. 100kDa.

Additional note: For Syntenin-1 in Hs745 and H596, only faint bands were observed. After re-probing the membrane was for GRP94 (and exposing for 1 min), bands of expected size of 32kDa approximately were observed. Bands of 32kDa (from (f)) were used to be in line with data for EBC1 (a)

S3. Images of non-cropped immunoblots from Figure 3c. Lane 1=Cell lysate, 2=UC-DG EV lysate, 3=ExoQuick EV lysate, 4= LSC EV lysate.

Evaluating a range of separation and characterisation methodologies to determine the relevance of extracellular vesicles as informers of the MET status of their cells of origin

(a)

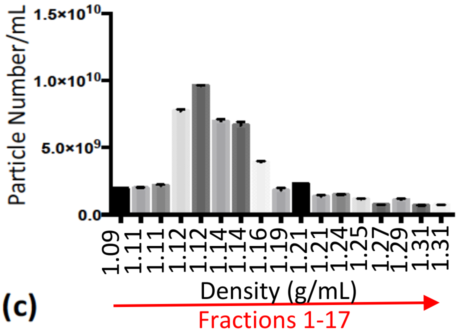

(b)

EBC1	Cell Number	Particle Number/mL
**Mean**	1.15E+07	5.24E+10
**Std. Deviation**	2.50E+05	4.36E+08
**Std. Error of Mean**	1.44E+05	2.52E+08

(c)

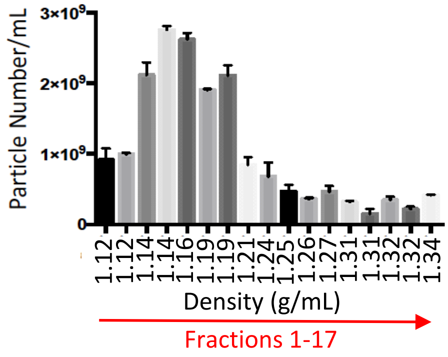

(d)

H596	Cell Number	Particle Number/mL
**Mean**	1.38E+07	1.77E+10
**Std. Deviation**	4.99E+05	1.40E+09
**Std. Error of Mean**	2.88E+05	8.09E+08

(e)

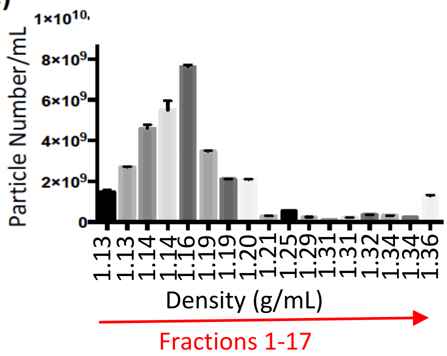

(f)

Hs746T	Cell Number	Particle Number/mL
**Mean**	1.23E+07	3.30E+10
**Std. Deviation**	2.50E+05	1.25E+09
**Std. Error of Mean**	1.44E+05	7.22E+08

S4. NTA of 17 fractions of EV containing samples following ultracentrifugation on density gradient. In order to determine the fractions containing EVs, particle concentration was measured by NTA and additionally, density of each fraction was measured using a refractometer (Atago). (a) The highest particle concentration (particles/mL) was recorded in EBC1 derived CM, in fractions 4-8; (c) Fractions 3-7 for H596 CM and (e) Fractions 2-8 for Hs746T CM. (b, d, f) indicate the total particle concentration across 17 fractions. Overall, samples containing the highest particle concentration were within density fractions of 1.12-1.2g/mL. Samples (fractions) containing the highest concentration of particles were pooled, washed with sterile particle-free PBS and centrifuged at 120,000g for 2 hours, in order to pellet EVs and to remove OptiPrep. NTA analysis was done on waste supernatant following 2-hour ultracentrifugation (in order to confirm that no particles were lost at the additional step of OptiPrep removal, data not shown). Data represented as the Mean of three biological repeats ± S.E.M.

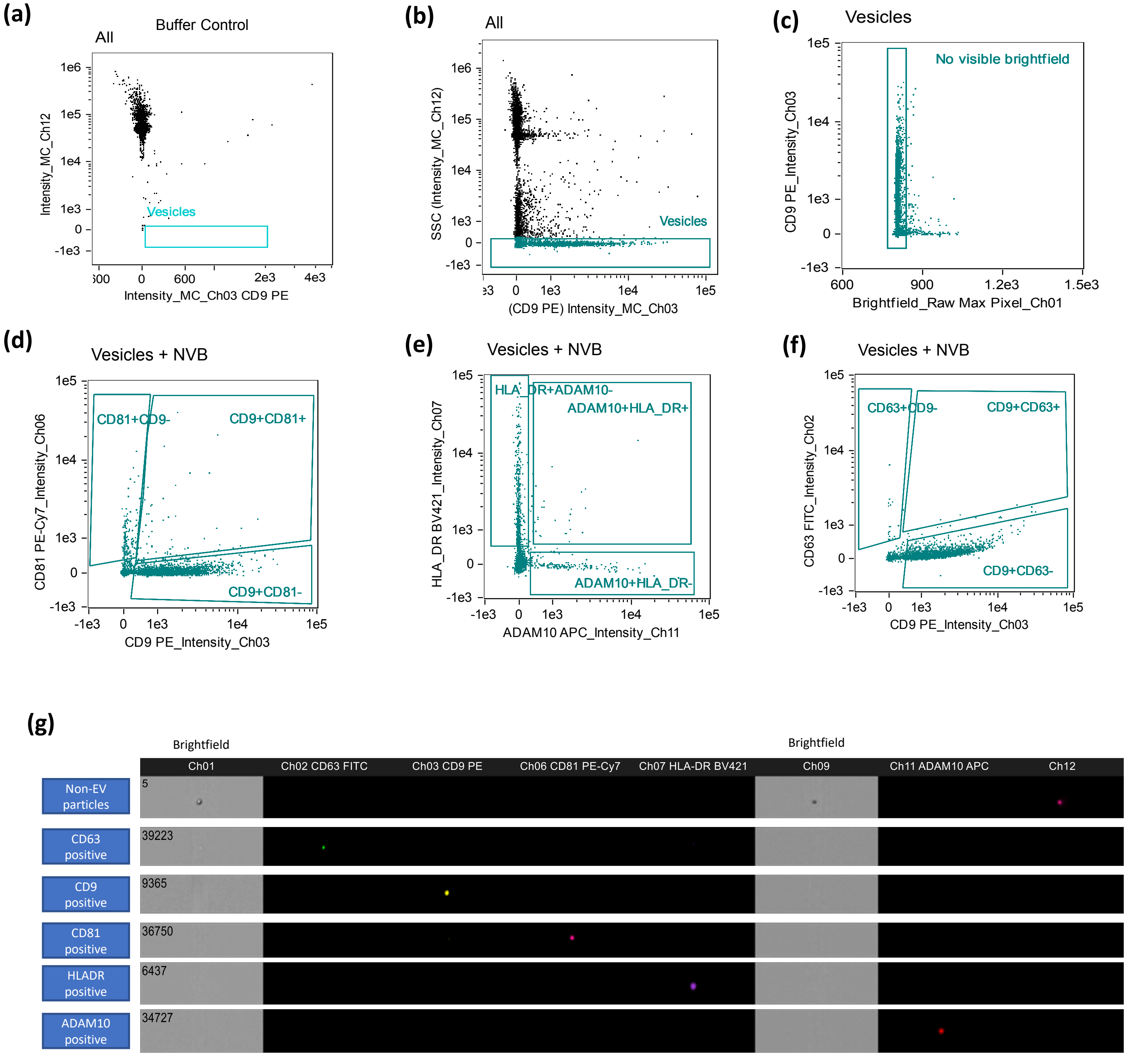

S5. Gating strategy and representative plots in EV identification. Vesicles (EVs) were acquired at 60x magnification, slow flow rate on an Amnis ImageStream^X Mark II Flow Cytometer. Controls without EVs were analysed to see possible background (a), EVs were first gated as side slcatter channel (SSC) low *vs* fluorescence (CD9 PE) (b), then as non-detectable brightfield (fluorescence *vs* Raw Max Pixel Brightfield channel) (c). (d-f) show representative plots of CD9 *vs* CD81 (d), ADAM10 *vs* HLA-DR (e) and CD9 *vs* CD63 (f). Representative image of EV protein expression (h).

(a)

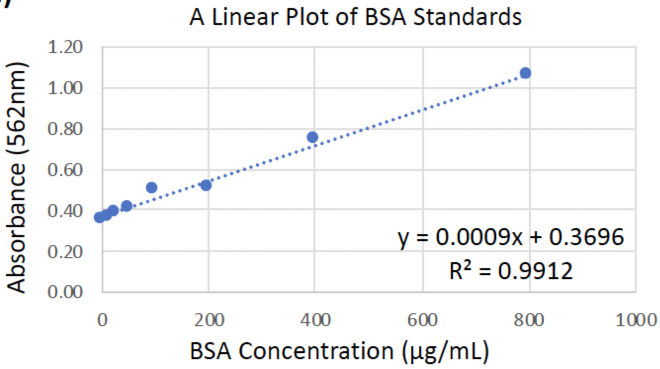

(b)

Sample	Protein (µg/µL)	Total Protein (µg)
**Cell Lysate**		
EBC1	57.30	11461.85
H596	63.08	12614.83
Hs746T	56.90	11382.23
**UC-DG**		
EBC1	2.96	443.53
H596	1.49	223.72
Hs746T	1.25	188.14
**ExoQuick**		
EBC1	3.43	1372.96
H596	2.47	989.70
Hs746T	1.63	650.00
**LSC**		
EBC1	1.40	210.56
H596	0.79	119.17
Hs746T	1.30	194.94

S6. Representative data of protein yields recovered from EVs separated by three different methods of isolation and their cells of origin. (a) A linear plot of BSA standards generated for extrapolation of protein concentration in EVs and their cells of origin. (b) Tabulated results of a typical protein yields obtained from cells collected from 2.5 x T175cm^2 culture flask (at 70% confluency), EV pellets following UC and LSC (EVs from 2.5 x T175cm^2 culture flasks) and ExoQuick (EVs in ExoQuick, separated from CM from 2.5 x T175cm^2 culture flasks).

Suppl. Table 1. Particle quantification by NTA

Cell Line	UC-DG		LSC		ExoQuick	
	**Mean**	**± S.E.M.**	**Mean**	**± S.E.M.**	**Mean**	**± S.E.M.**
**EBC1**	8.63E+10	2.57E+09	2.08E+10	4.10E+08	7.71E+08	1.15E+08
**H596**	6.95E+10	4.52E+09	1.14E+10	6.96E+08	5.71E+09	1.20E+09
**Hs746T**	2.90E+10	1.03E+09	1.16E+10	2.06E+09	3.74E+09	2.26E+09

Tabulated results for Figure 2a, showing Mean ± S.E.M of ≥n=3 repeat analysis on samples from n=3 biological repeats

Suppl. Table 2. The percentage of EVs positive for a range of EV markers

Cell Line	CD63+	CD9+	CD81+	ADAM10+	HLA-DR+	Other EVs
**EBC1**						
UC-DG	3.02	38.41	2.84	1.29	5.91	48.53
LSC	1.95	33.37	3.57	1.74	13.53	45.83
ExoQuick	2.04	36.35	3.57	1.99	10.27	45.76
**H596**						
UC-DG	2.64	19.21	11.40	2.51	33.44	30.79
LSC	2.75	20.41	15.04	3.36	27.34	31.10
ExoQuick	3.16	26.90	9.35	2.05	34.23	24.31
**Hs746T**						
UC-DG	3.01	24.25	8.53	1.32	38.47	24.41
LSC	3.86	26.24	7.01	1.44	44.02	17.44
ExoQuick	3.06	21.30	13.32	3.35	26.00	32.98

Tabulated results for Figure 3b

Suppl. Table 3. Total RNA yield in EV samples from different methods of EV isolation and corresponding particle counts used to produce total RNA yield

Sample	RNA Yield (μg)	± S.E.M.	Particle Count	± S.E.M.
**EBC1**				
UC-DG	8.86	0.46	2.16E+09	6.44E+07
LSC	5.49	0.55	1.74E+09	1.13E+08
ExoQuick	2.85	0.07	7.25E+08	2.56E+07
**H596**				
UC-DG	8.74	0.27	5.21E+08	2.87E+06
LSC	4.60	0.17	2.84E+08	3.00E+07
ExoQuick	2.65	0.10	2.91E+08	5.65E+07
**Hs746T**				
UC-DG	5.68	0.17	1.93E+07	1.02E+07
LSC	5.17	0.19	1.43E+08	1.74E+07
ExoQuick	3.16	0.32	9.35E+07	5.16E+07

Tabulated results for Figure 4

Suppl. Table 4. Protein yield in EV-lysates from different methods of EV isolation and their corresponding cell lysates

Cell Line	Sample	Mean Yield (µg)	± S.E.M.
**EBC1**	Cell Lysate	4919.33	226.98
	UC-DG	411.00	17.58
	LSC	202.50	18.99
	ExoQuick	1362.67	40.88
**H596**	Cell Lysate	4736.00	364.29
	UC-DG	235.50	7.94
	LSC	133.00	9.73
	ExoQuick	1201.33	122.41
**Hs746T**	Cell Lysate	5308.67	690.75
	UC-DG	189.50	5.29
	LSC	192.50	4.09
	ExoQuick	649.33	34.67

Tabulated results for Figure 5
